# Shifting seasonality of cyclones and western boundary current interactions in Bay of Bengal as observed during Amphan and Fani

**DOI:** 10.1038/s41598-021-01607-6

**Published:** 2021-11-11

**Authors:** Sourav Sil, Avijit Gangopadhyay, Glen Gawarkiewicz, Saikat Pramanik

**Affiliations:** 1 School of Earth, Ocean and Climate Sciences, Indian Institute of Technology, Bhubaneswar, India; 2 School for Marine Science and Technology, University of Massachusetts Dartmouth, Dartmouth, MA 02747 USA; 3 Woods Hole Oceanographic Institution, Woods Hole, MA 02543 USA

**Keywords:** Physical oceanography, Climate change, Natural hazards, Atmospheric dynamics

## Abstract

In recent years, the seasonal patterns of Tropical Cyclones (TC) in the Bay of Bengal have been shifting. While tropical depressions have been common in March–May (spring), they typically have been relatively weaker than the TCs during October–December. Here we show that the spatial pattern of recent warming trends during the last two decades in the southwestern Bay has allowed for stronger springtime pre-monsoon cyclones such as Amphan (May 2020, Super Cyclone) and Fani (April–May 2019, Extremely Severe Cyclone). The tracks of the pre-monsoon cyclones shifted westward, concurrent with an increasing rate of warming. This shift allowed both Fani and Amphan tracks to cross the northeastward warm Western Boundary Current (WBC) and associated warm anti-cyclonic eddies, while the weaker Viyaru (April 2013, Cyclonic Storm) did not interact with the WBC. A quantitative model linking the available along-track heat potential to cyclone’s intensity is developed to understand the impact of the WBC on cyclone intensification. The influence of the warming WBC and associated anti-cyclonic eddies will likely result in much stronger springtime TCs becoming relatively common in the future.

## Introduction

Tropical Cyclones are one of the most devastating natural disasters, especially over coastal regions, due to impacts on densely populated low-lying areas and conditions over shallow continental shelves that strengthen Tropical Cyclone intensity^1^. In recent years, the increasing trend in the intensification rates of Tropical Cyclones has been observed on a global scale^2^.

The Bay of Bengal, a unique tropical ocean basin in the northern Indian Ocean, is a potentially active region for the genesis of Tropical Cyclones. Formation of Tropical Cyclones over this region is seasonal with a primary peak formation during the post-monsoon season (October–December) and a secondary peak formation during the pre-monsoon season (March–May)^3^. The role of atmospheric parameters and sea surface temperature (SST) in cyclone formation and intensification are well established from the satellite and available in-situ observations during the different cyclonic events^4–10^. The SST, the dynamic topography, and the presence of oceanic eddies at the surface all have a significant contribution to Tropical Cyclone intensification^5^. Surface eddies are strongly related to the variability of winds and sea surface height anomalies (SSHA) in the Bay of Bengal^11,12^. Warm-core eddies (with positive SSHA) have a deeper thermocline, which supplies significant heat for cyclone intensification. In contrast, cold-core eddies (with negative SSHA) weaken cyclone intensity due to a shallower thermocline and significantly less heat content^11,12^.

**Figure 1:**
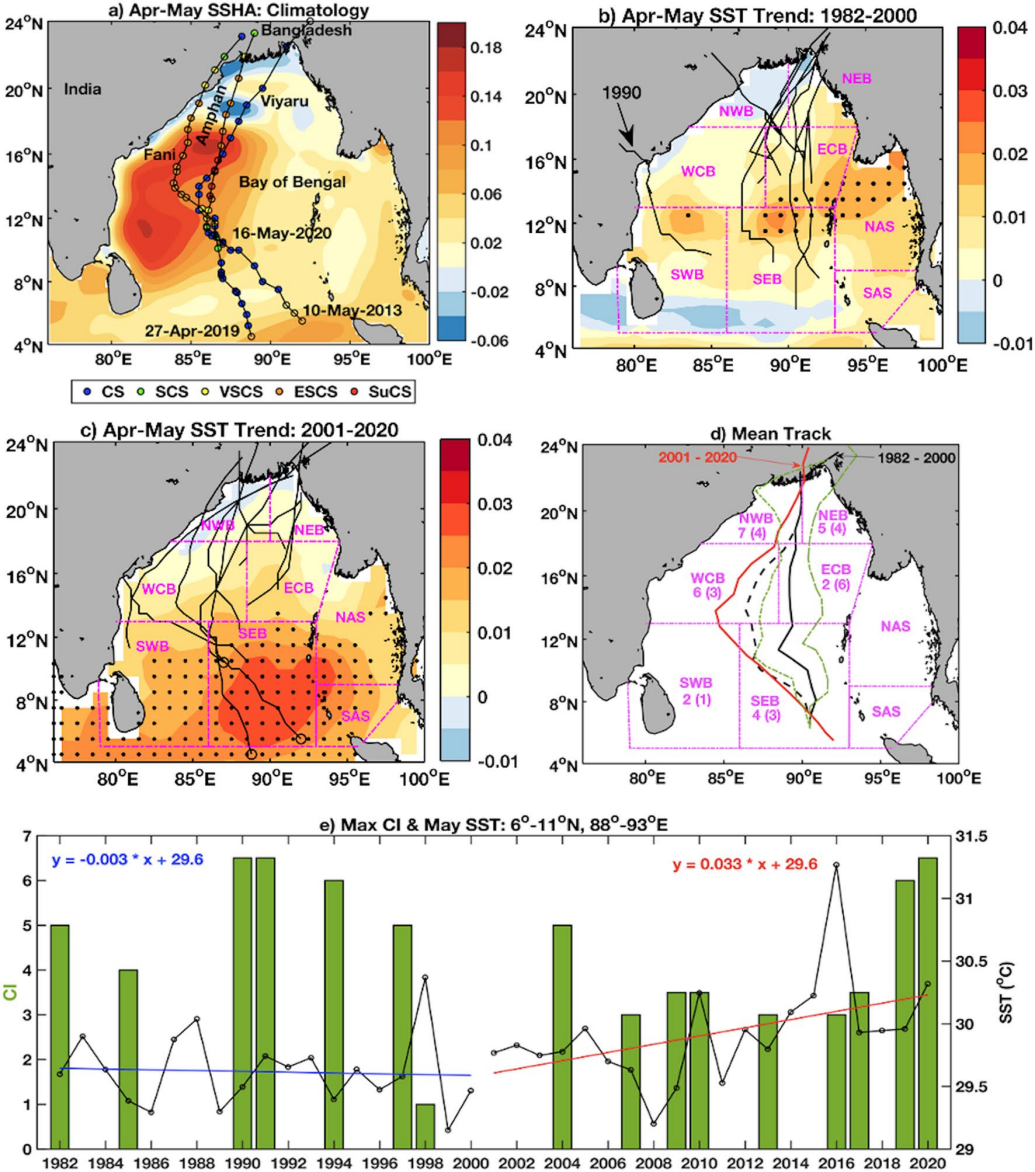
(**a**) Best-estimate tracks for cyclones Viyaru (10th–16th May 2013), Fani (27th April–3rd May 2019), and Amphan (16th–21st May 2020). Different stages of development for each cyclone are shown along each track with color filled circles, which are explained in the legend below the panel. Climatological SSHA for April–May in the background shows the typical coverage of the WBC and its warm eddies during spring. (**b**) The SST trend ($^\circ \text{C}/\text{year}$) during April–May and cyclone tracks during 1982–2000 are shown. (**c**) Similar to (**b**), but for 2001–2020. The magenta boundaries show the regions of the Bay of Bengal as per IMD (see “Methods” section). The shaded ‘$\cdot$’ regions in (**b,c**) show 90% significance of the SST trend in their respective periods indicating the southwestward shift of the same in recent decades. (**d**) Mean tracks of the pre-monsoon cyclones (except the 1990 cyclone with land fall around 16$^\circ\, \text{N}$) during 1982–2000 (solid black) and during 2001–2020 (red line). The dashed black line denotes the mean track including the 1990 cyclone. The dotted green lines are the one standard deviation of cyclones (except 1990). Westward shifting of the Pre-monsoon cyclone tracks is evident. The numbered pairs ‘m (n)’ indicate the number of cyclones passing through each IMD region during ‘2001–2020 (1982–2000)’. (**e**) Maximum current intensity (CI) (bar diagram) and SST yearly trend during 1982–2000 (blue line) and 2001–2020 (red line) in the southern Bay of Bengal (6–11$^\circ\, \text{N}$, 88–93$^\circ\, \text{E}$). The maps (coastlines) are created with Matlab M_Map v1.4 toolbox (https://www.eoas.ubc.ca/~rich/map.html).

The oceanic circulation in the spring or pre-monsoon season is dominated by the northward flowing warm western boundary current (WBC) and its eddies^13,14^. Formation of the WBC is due to the anti-cyclonic wind gyre that develops in November and continues through April–May by the integrated wind stress curl or Ekman pumping^15,16^. The WBC propagates northward from 10 to 17° N along the edge of the continental shelf and then separates at around 18° N and flows eastward^13^ with multiple eddies (Fig. 1a). This springtime WBC is observed 50–100 km away from the coast with a width of 200–300 km and extending down to depth of 250–450 m^17,18^ before separating from the coast with associated warm eddies. It is typically associated with positive SSHA with values from 0.1 m onshore to 0.4 m offshore with current magnitude of about 1–1.8 m s^−1^^19,20^.

Recently, the Bay of Bengal has experienced rapid intensification (defined as an increase of at least 30 knots in wind speed over a 24 h period)^21,22^ of an Extremely Severe Cyclonic Storm (ESCS) Fani during 27 April–3 May 2019 and another Super Cyclonic Storm (SuCS) Amphan during 16–21 May 2020 as per India Meteorological Department (IMD). After 1991, Amphan was the first SuCS in twenties century to appear in the Bay of Bengal during pre-monsoon season. According to the Joint Typhoon Warning Center (JTWC) real-time data, these cyclones are categorized as Category 4 and 5, respectively. Cyclone Fani rapidly intensified from Severe Cyclonic Storm (SCS) to ESCS during 29–30 April 2019. The wind speed increased from 55 to 90 knots in 24 h. Cyclone Amphan rapidly intensified from SCS to SuCS during 17–18 May 2020, when the wind speed increased from 55 to 120 knots in 24 h. In contrast, another Cyclonic Storm (CS) Viyaru (tropical storm as per JTWC) during May 2013, which followed a similar initial track to Fani, was much more limited in intensity. Both Fani (2019) and Amphan (2020) formed in the western part of southeastern Bay of Bengal and interacted with the western boundary current, while Viyaru veered to the east of the WBC region (tracks shown in Fig. 1a). The descriptions of these three cyclones are given in the Methods section.

Recent studies show that there has been little significant changes in the atmospheric conditions vertical shear and humidity; however, the oceanic parameters SST and ocean heat content have increased in the recent decades in the Bay of Bengal^23–25^. The frequency of the cyclones did not change much; however, the cyclones have become more intensified due to the warming of the SST^23,26,27^. On the other hand, the intraseasonal variability in the met-ocean parameters might have caused the observed rapid intensification of the cyclones. For example, in case of Fani, the rapid intensification was partially due to the convective coupling of atmospheric Kelvin waves from the upper troposphere with the Madden Julian Oscillations (MJO)^25,28^. Another study showed positive phases of intraseasonal variability of SST at the equatorial Indian Ocean modulate the northward shifting of the cyclogenesis locations in the Indian Ocean from tropical Indian^29^. On the contrary, cyclone Viyaru did not intensify probably because the development of the lower tropospheric and upper tropospheric potential vorticity was low and quasi-static during the lifecycle of the cyclone Viyaru^30^. In the case of Amphan, Bhowmick et al.^31^ showed from the buoy observations that under low wind speed conditions and the warm oceans in early May heated the atmosphere by enhancing the long wave radiation from the atmosphere to the ocean. They suggested to consider the ocean and associated air–sea interaction processes that take place prior to cyclogenesis. However, the role of the ocean, particularly that of the seasonally occurring northward warm WBC on the spring cyclones in the Bay of Bengal, has not been fully explored yet.

The increased severity of springtime pre-monsoon cyclones in the recent past, including the successive ones in 2019 and 2020, led us to hypothesize and investigate (i) the possibility of linkage between the recent observed warming in the Bay of Bengal and the genesis of these cyclones; and (ii) if the answer to (i) is a yes, then to further investigate the second question: Why was it that Viyaru in 2013, which started at a very similar location as Fani in 2019, and followed a very similar path initially, ended up becoming a CS, while Fani reached a maximum strength of the stage of ESCS? We hypothesize that the oceanic circulation, particularly the heat content from the northward flowing WBC and its eddies, might substantially affect the temporal evolution of intensity of pre-monsoon Tropical Cyclones in the Bay of Bengal during spring. We present the results of our investigation to address the two hypotheses next.

### Shifting seasonality (stronger pre-monsoon cyclones due to warming SST trends)

During 1982–2020, a total of 77 cyclones formed over the Bay of Bengal with landfall in India and the Bangladesh coast (source: IMD cyclone e-atlas^32^). Of these, 19 (25%) formed in the pre-monsoon (March–May) season and 58 in the post-monsoon (October–December) season. The formation locations and subsequent tracks of the 8 pre-monsoon cyclones prior to 2000 and 10 pre-monsoon cyclones after 2000 are shown in Fig. 1b,c. Note that one pre-monsoon cyclone in 1989 could not be considered due to unavailability of its data. The IMD-recognized sub-regions are also shown in Figs. 1b–d. During 1982–2000, most of the pre-monsoon cyclones formed over the east-central (ECB) and southeastern (SEB) Bay of Bengal (Fig. 1b). Six out of eight tracks passed through the ECB and northern Bay of Bengal. Therefore, they did not interact with the western boundary current, which extends to 88$^\circ\, \text{E}$ in the northern bay between 18 and 20° N and is limited to 85$^\circ\, \text{E}$ between 8 and 10$^\circ\, \text{N}$. In this study, we consider 0.15 m of SSHA to represent the western edge of the WBC (Fig. 1a and subsequent figures). All of these cyclones made landfall to the north of 20$^\circ\, \text{N},$ except one in 1990, which made landfall at around 16$^\circ\, \text{N}$ and crossed the WBC, and became a super cyclone.

Most of the subsequent pre-monsoon cyclone tracks have exhibited a distinct westward shift during 2001–2020 (Fig. 1c) compared to their 1982–2000 path. In the recent decades, six out of ten cyclones moved through the west-central bay B) and seven through northwestern bay (NWB). Therefore, the chance of their interaction with the warm WBC associated with positive sea surface height has increased. The mean track during 1982–2000 starts near the central part of SEB and makes landfall over Bangladesh (Fig. 1c). The dashed black line shows the mean track while including the anomalous 1990 cyclone, which crossed over the WBC region and became a super cyclone. The mean longitude and latitude of the genesis location in the earlier decades (1982–2000) was 89.6$^\circ$ E, 11.6$^\circ$ N, and in the recent decades (2001–2020), it is 87.9$^\circ$ E, 13.9$^\circ$ N. Comparison of the mean tracks shows that the westward shifting is maximum in the central Bay of Bengal. Additionally, this westward shifting of tracks is more than one standard deviation (green dash line, Fig. 1d) of the 1982–2000 path in the southern and central Bay of Bengal. The current intensity (CI), a measure of the instantaneous cyclone strength (see “Methods” section) showed a downward trend from 1982 to 2000 and an upward trend after 2000 (green bars, Fig. 1e). The CI varies from 1.5 to 7 as provided by IMD best track data^33,34^ (see “Methods” section). Together with the comparison of mean formation region and subsequent mean tracks in the two periods, it is evident that there has been a significant westward shift in the genesis location and the post-2000 pre-monsoon cyclones are moving through a more westward path which increases their chances of interaction with the warm springtime WBC in the Bay of Bengal.

The possible impact of SST and its trends have been shown to affect the intensity of cyclones in recent studies in the Indo-Pacific region^35,36^. To understand this relationship between the formation regions and the tracks with the SST, the trends of SST over the Bay of Bengal were computed for different seasons using the Hadley SST^37^ (see “Methods” section). The SST in the WBC region is generally 1–2 °C warmer than the central basin associated with the positive SSHA (Fig. 1a) and anticyclonic eddies^13,14^. The SST trends in April and May are positive in the east-central (during 1982–2000) and southern regions (during 2001–2020) of the Bay of Bengal. A student’s two-tail t-test was performed which identified the SST trends with significance level more than 90% during 1982–2000 and 2001–2020. These regions are marked by ‘+’ signs in Fig. 1b,c showing the southwestward movement of the formation of cyclone genesis in the later decades. Note that the SST trend for the latter period is with higher positive values (0.02–0.04 °C per year) and extended over a larger region in the southern Bay of Bengal. The trend for the earlier two decades (1982–2000) did not show appreciable regions of significant warming (trend value 0–0.015 °C per year), indicating the robustness of the recent changes observed in the SST trend.

**Figure 2:**
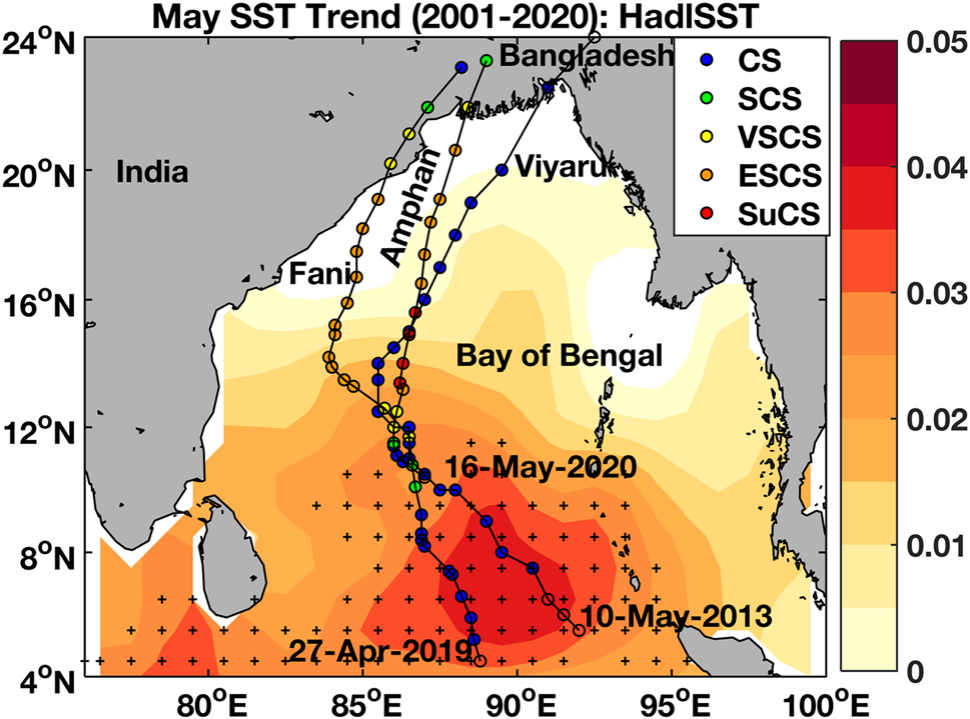
The background is the warming trend ($^\circ{\text C}$/year in colorbar) of SST during May. Note that all three recent cyclones utilized the warming in the southeastern Bay of Bengal to intensify, although Viyaru was limited in intensity by its more eastward track. The shaded ‘+’ regions show 95% significance of the SST trend. The map (coastlines) is created with Matlab M_Map v1.4 toolbox (https://www.eoas.ubc.ca/~rich/map.html).

A large number of cyclones (15) out of the total 19 pre-monsoon cyclones during 1982–2020 formed in the Bay of Bengal during the month of May. Among the other four cyclones, three including Fani, formed in late April and made landfall in early May. This motivated us to consider three recent cyclones in recent times (2013–2018) for May. There were other cyclones between 2013 and 2018 (Fig. 1d), for example, in 2016 and 2017; however, they did not attain the stage of VSCS, which was done by both Fani and Amphan in succession. We also compare the ocean features for Viyaru as it formed on the southeast part of Bay of Bengal similar to Fani and Amphan, but did not intensify to a stage more than CS stage. We first analyze the SST trend separately for May during 2001–2020. The average SST trend in the Bay of Bengal (4–24$^\circ\, \text{N}$, 76–100$^\circ\, \text{E}$) in May is 0.01 ${^\circ{\text C} }$ per year for the duration 1982–2020. The trend is higher (0.016 ${^\circ{\text C} }$ per year, 95% significance) in the 2001–2020 compared to 1982–2000 (0.004 ${^\circ{\text C} }$ per year, 90% significance) (Figure not shown). The warming trend is higher in the Bay of Bengal in comparison to the global mean trend of 0.006–0.01 ${^\circ{\text C} }$ per year^38^. These warming trends calculated here are similar to those reported by Zhang et al.^35^ (see their Fig. 1) and Thanh et al.^36^ (see their Fig. 7) for the Indo-Pacific tropical region. The formation regions of Tropical Cyclones in the last two decades are aligned with the maximum SST trend region (0.02–0.04 °C per year, 95% significant) depicted by the southeast-to-northwest elongated core structure (4–12° N, Fig. 2) in the southeastern Bay of Bengal, where the recent Tropical Cyclones Viyaru, Fani and Amphan were all formed. Interestingly, the northern part of this core is only observed in the May trend (Fig. 2) in contrast to the April–May trend (Fig. 1c). Note that the Amphan cyclone rapidly intensified into a super cyclone while passing over the northern part of this core. In 24 h (17th May 0600 UTC to 18th May 0600), it became a super cyclonic storm (13.4$^\circ\, \text{N}$, 86.2$^\circ\, \text{E}$) from severe cyclonic storm (11.5$^\circ\, \text{N}$, 86$^\circ\, \text{E}$). On the other hand, cyclone Fani, which formed in late April, did not get the benefit of passing through the northern core structure, and was limited to a weaker ESCS status (albeit, similarly devastating to Amphan due to its inshore track) before landfall. The SST trend in the southern Bay of Bengal (6–11$^\circ\, \text{N}$, 88–93$^\circ\, \text{E}$) also showed a remarkable warming trend of 0.033 $^\circ{\text C}$ per year from 2001 to 2020, which was almost negligible from 1982 to 2000. The changes in the SST in last two decades also match with the increasing changes of the CI of the cyclones (Fig. 1d).

### Quantification of the impact of the WBC and its associated eddies on the cyclone intensity

While the answer to the first question appears to be affirmative (SST warming trend is partially and significantly responsible for relatively westward springtime cyclone genesis in recent two decades), the answer to the second question lies in the possibility of the cyclone’s path crossing the WBC and its anticyclonic eddies, thereby gathering energy. This interaction has happened for Fani and Amphan, and not for Viyaru. A simple quantitative model of computing the “along-Track Available Potential Heat” (TRAPH) or the heat potential under the footprint of the evolving cyclone’s stronger wind region is developed first. Relating this TRAPH with the current intensity (CI) will then be used to examine the relationship between the ocean heat content and the evolution of the cyclone intensity.

The TRAPH is calculated in four distinct steps. First, wind speed around the center of the cyclone is analyzed to obtain a typical radius of influence for the cyclone’s energy. It is typically considered as the distance from the center where the wind speed reaches its maximum and then decreases. For all three cyclones, this distance was determined from the zonal and meridional variation of the wind speed along the radials from the center of the cyclone. However, as the cyclone evolves along its path, the wind speeds are not radially symmetric. There are patches of stronger winds, or footprints of the high-energy bands of the cyclone. These are caused by complex non-linear processes of multi-layer atmospheric flow, driven by advection, convection, precipitation and interaction with the ocean^39,40^. Thus, in the second step, we choose a threshold wind speed, where the maximum sustainable winds occur. The threshold values are considered close to the 90th percentile of the winds over the Bay of Bengal for three cyclones. See “Methods” section for details on threshold calculation. This threshold wind speed was chosen as 15 m s^−1^ for the SuCS (Amphan), and 12 m s^−1^ for ESCS (Fani) cyclones. For Viyaru, which reached only to the stage of cyclonic storm, this speed was chosen as 10 m s^−1^. In the third step, the region within the radius of influence that had wind speeds more than the threshold was demarcated as the “energy gathering footprint” of the cyclone. This is the effective region within the cyclone where the winds gather energy from the ocean. In the fourth and final step, the TRAPH is determined as the oceanic heat content within the active footprint of the cyclone using the SST distribution from the first day of the cyclone initiation as follows:

$$ TRAPH = \rho C_{p} H \mathop{{\int\!\!\!\!\!\int}\mkern-21mu \bigcirc} {T\left( {x,y} \right) dx dy} , $$

where ${C}_{p}$ is the specific heat (4.2 $\times$ 10^3^ J kg^−1^ K^−1^), $\rho$ is the sea water density (1025 kg m^−3^), $H$ is the mixed-layer depth, and $T\left(x,y\right)$ is the SST distribution. $H$ is assumed to be constant at 50 m^41–43^ for this simple calculation. Available Argo profile (WMO: 2902283) in central Bay of Bengal around 84.3$^\circ$ E, 14.85° N during May 2020 also confirmed a mixed-layer depth of 50–60 m. The area integral is performed for the footprint region only. TRAPH is interpolated at 6-hourly intervals from the daily SST and wind fields obtained from satellites (see “Methods” section for sources).

**Figure 3:**
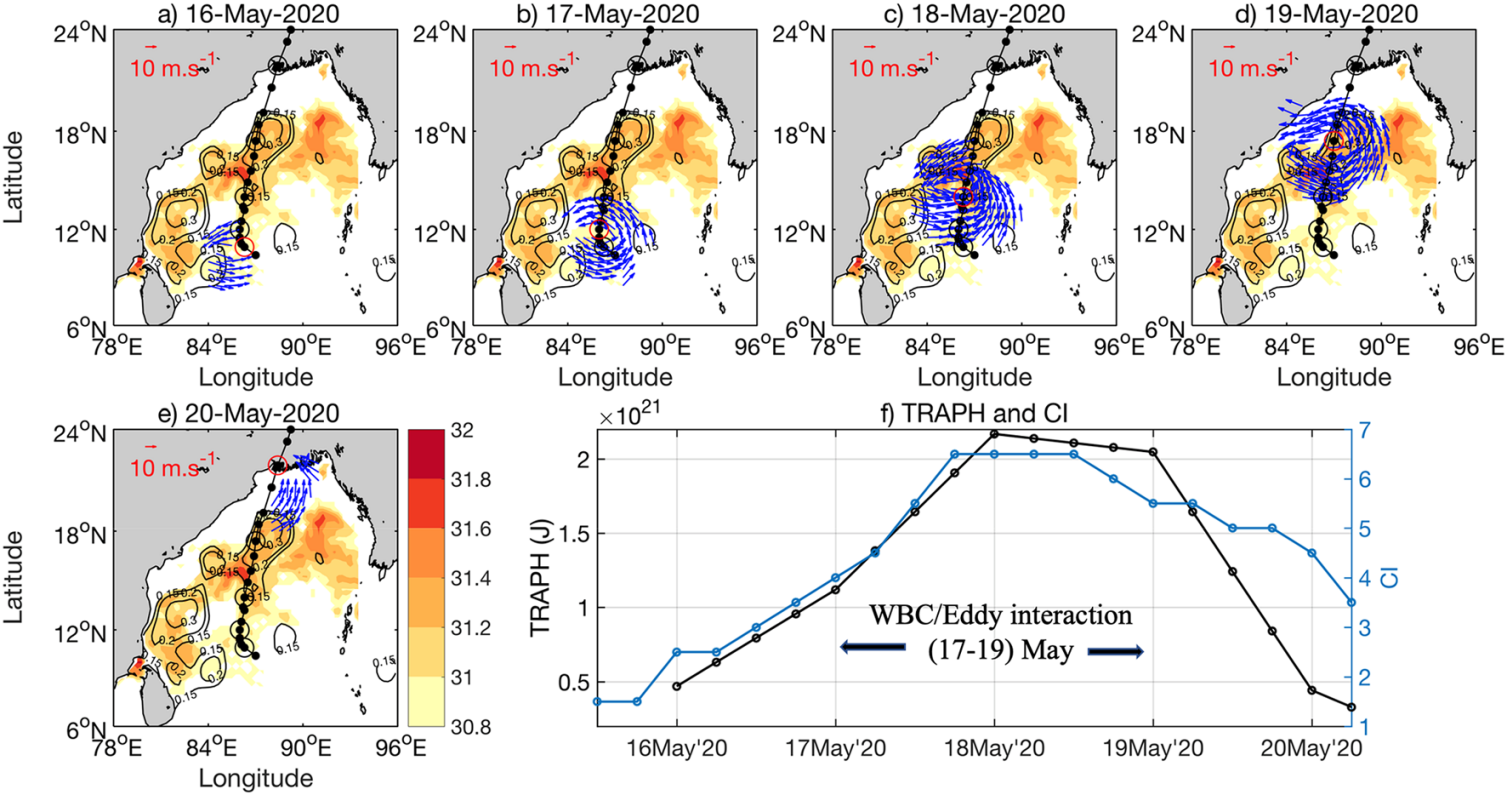
(**a–e**) Evolution of cyclone Amphan during 16th, 17th, 18th, 19th, and 20th May 2020, respectively. Background shaded SST and SSHA (contours) are for 15th May 2020 (for SST > 30.8 $^\circ{\text C}$). The SSH contours in black show the WBC and the three anticyclonic eddies (high SSH values of 0.15 m). The blue wind vectors are for wind speeds more than 15 m s^−1^. The black dots along the tracks represent the position of the cyclone center. (**f**) The two lines in the right-bottom panel are: (i) blue for CI of cyclone and (ii) black for the representative heat index (in Joule) available for cyclone intensification (TRAPH). See text for details. The black dots indicate the location of cyclone every 6 h. The black open circles indicate the positions of the Cyclone on each day (at 1200 UTC) during its whole journey. The red circle in each panel shows the location of the cyclone on that particular day at 1200 UTC. The interaction period between the cyclone with the WBC and its warm anticyclonic eddies are indicated by the horizontal arrows. The maps (coastlines) are created with Matlab M_Map v1.4 toolbox (https://www.eoas.ubc.ca/~rich/map.html).

**Figure 4:**
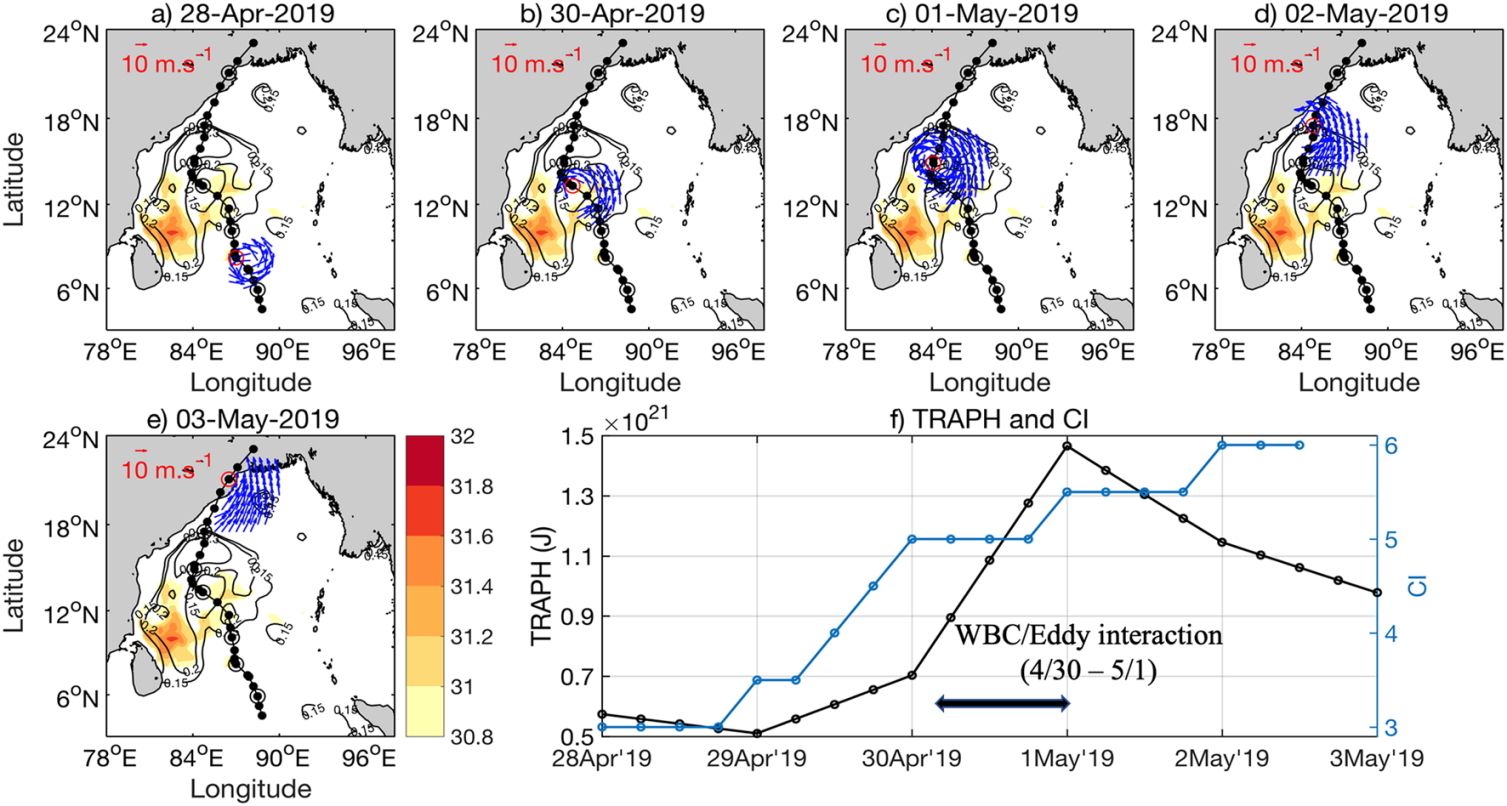
(**a–e**) Similar to Fig. 3a–e, but for Fani. Background shaded SST and SSHA (contours) are for 26th April 2019 (for SST > 30.8 $^\circ{\text C}$). Evolution of cyclone Fani is shown with SST, SSH and Winds for 28th April, 30th April, 1st May, 2nd May, and 3rd May 2019. (**f**) Similar to 3f. but for Fani. Note the similarity of heat availability following CI in the bottom right panel. See text for details. The maps (coastlines) are created with Matlab M_Map v1.4 toolbox (https://www.eoas.ubc.ca/~rich/map.html).

**Figure 5:**
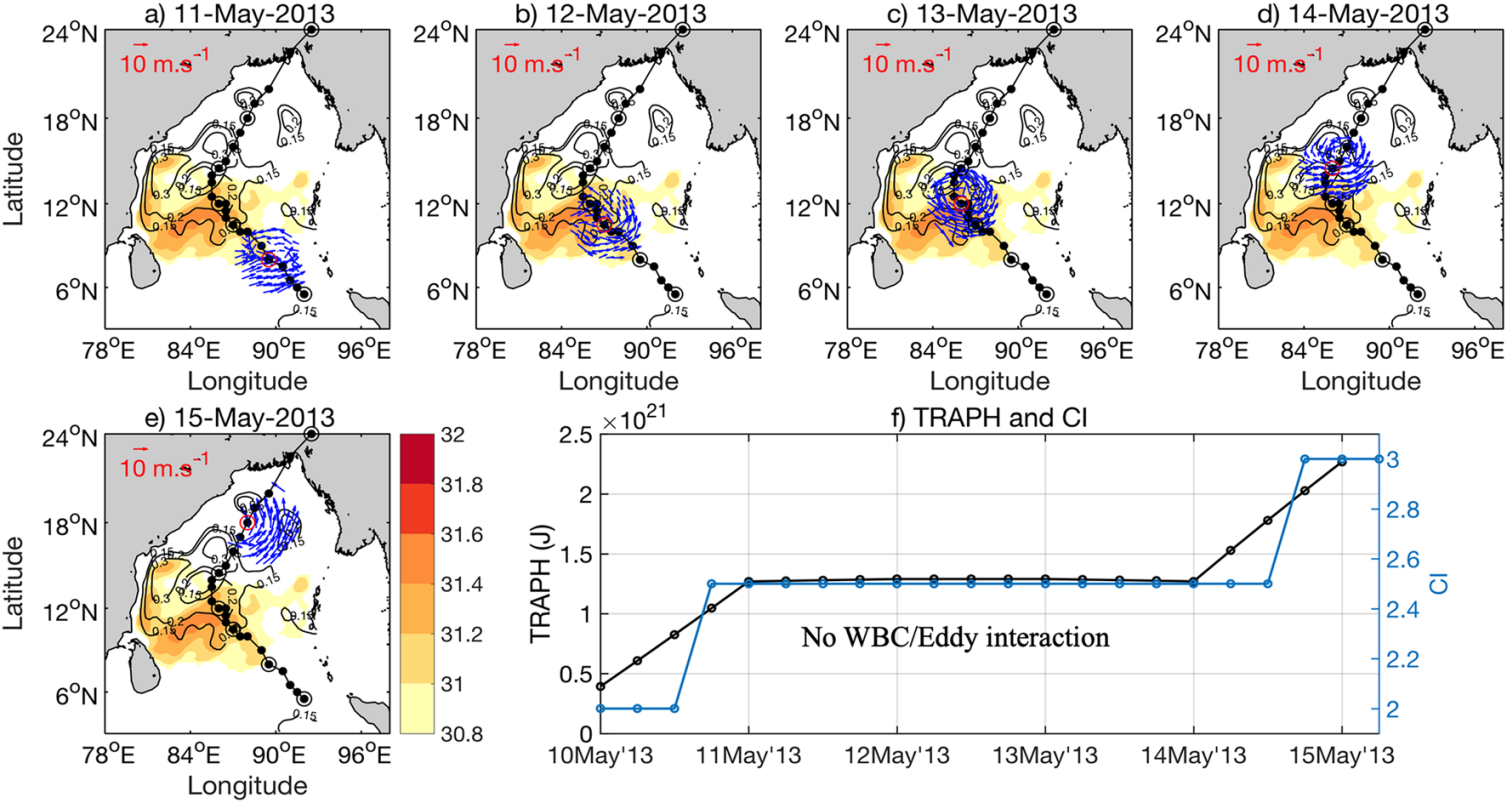
(**a–e**) Similar to (a–e) in Figs. 3 and 4, but for Viyaru (2013). Background shaded SST and SSHA (contours) are for 9th May 2013 (for SST > 30.8 $^\circ{\text C}$). Evolution of cyclone Viyaru is shown with SST, SSH and Winds for May 11–15, 2013. (**f**) Similar to Figs. 3f and 4f, but for Viyaru. Note how the CI and Heat availability, in this case is flat during most of its evolutionary path (bottom right panel) that prevented strengthening. The maps (coastlines) are created with Matlab M_Map v1.4 toolbox (https://www.eoas.ubc.ca/~rich/map.html).

Figures 3, 4 and 5 present the evolutions of the three cyclones, Amphan, Fani and Viyaru, respectively. We choose Amphan, Fani and Viyaru to describe the application of the above quantitative model. The case for Amphan is like Fani, except that it crossed over two anticyclonic eddies and the retroflected large anti-cyclonic warm eddy-like feature of the eastern segment of the separated WBC (87.2$^\circ$ E, 18.4° N) (see Fig. 3b–d). Amphan moved over the WBC region and its warm eddies (see the 0.15 m iso-height contours) during 17–19 May 2020 while reaching the super cyclonic stage on 18 May, 0600 UTC, and becoming a ESCS on 19 May, 0600 UTC. This interaction period is indicated within Fig. 3f. Fani crossed over the WBC and its warm eddies during 30 Apr to 02 May 2019. Viyaru, on the other hand, was over a cyclonic eddy on 14 May 2013 and became stagnant, probably losing some of its heat. The tropical cyclone Viyaru veered to the right and missed interacting with the WBC eddies to its west. Also note that the WBC and its eddies were situated further to the east during 2019 and 2020 (88$^\circ$ E) than in 2013 (90$^\circ$ E). This allowed the recent cyclones (Fani and Amphan) to gather more heat and strengthen while passing over the eddies. On the other hand, Viyaru did not interact with the WBC and its eddies in 2013 and could not strengthen beyond the stage of CS.

Eddy passage by a Tropical Cyclone is one of the most crucial parameters determining the evolution of the storm intensity. Cyclones intensify after passing over an anticyclonic (warm) eddy with positive SSHA, and dissipate when it is crossing over a cyclonic (cold) eddy with negative SSHA^4,5^. Climatologically, during the pre-monsoon season (April–May), a strong anti-cyclonic gyre dominates over the western Bay of Bengal and extends from 7 to 18° N ^12,17^. Both Amphan (Fig. 3a–e) and Fani (Fig. 4a–e) passed close to strong positive SSHA anomalies and had generally northward tracks. However, Viyaru (Fig. 5a–e) curved to the northeast near 14° N, moving the Tropical Cyclone well away from the strong positive SSHA near the continental shelf. The proximity to the WBC and anti-cyclonic eddy likely boosted the intensity of Amphan and Fani, while the northeastward track of Viyaru likely was the primary factor limiting its intensity to CS.

It is evident from the three bottom-right panels of Figs. 3, 4 and 5 that the TRAPH follows and leads the current intensity (CI) reasonably well for all three cyclones. For Fani and Amphan, this quantitative pattern provides support for the hypothesis that indeed these two severe cyclones reached their peak after interacting with the WBC and its associated anticyclonic warm eddies. Figure 5 also indicates that since Viyaru did not interact with the WBC and its eddies, it did not gather strength after becoming a CS in the first 2–3 days and maintained its stage of CS throughout its journey.

The synoptic distribution of GHRSST (Global High Resolution Sea Surface Temperature) for all three cyclones are shown in the color background field of Figs. 3, 4 and 5. During the passage of Fani in April–May 2019, the northward propagating WBC was strong, extending northward to 18$^\circ\, \text{N}$, and then bifurcating to form the cyclonic eddy near 18 $^\circ N$ over the northern Bay of Bengal (Fig. 4). On 30th April 2019, the center of Fani was over a warm temperature region (> 30.8 °C) in the west-central Bay of Bengal (Fig. 4b). While temperatures generally decreased to the north, the presence of an anti-cyclonic eddy kept temperatures warmer in the western Bay of Bengal (Fig. 4a–e). Note that the area of the higher SST (for SST > 30.8 ℃) is much larger in case of Amphan than that of Fani. In contrast, during the passage of Viyaru in May 2013, the WBC only extended to 16$^\circ\, \text{N}$ before moving northeastward away from the coast (Fig. 5; see contour of SSHA = 0.15 m).

Similarly, for Viyaru, initially, SST was higher in the central Bay of Bengal (Fig. 5a,b) and SST decreased with the northward movement of the cyclone center (Fig. 5c–e). However, because of the much more southerly detachment point of the western boundary current, Viyaru encountered substantially cooler waters much further south than Fani encountered. Note that the decline of TRAPH for all three cyclones nearing the end of the time-series in Figs. 3f, 4f and 5f is a signature of the post-storm cooling, which is a function of the rapidly diminishing CI of the cyclone^44,45^.

Historically, most of the cyclones in the Bay of Bengal occur during the fall-autumn post-monsoon season. Recent warming in the Indian Ocean has seemingly resulted in cyclone developments in May 2013 (Viyaru, CS), April–May 2019 (Fani, ESCS) and in May 2020 (Amphan, SuCS). According to the reports of IMD (2013, 2019, 2020), Fani^46^ became an Extremely Severe Cyclone (maximum sustained wind speed (MSWS) of 59 m s^−1^, estimated central pressure (ECP) of 920 hPa, and central pressure drop (CPD) of 66 hPa) over the ocean. Amphan reached a SuCS stage (MSWS of 67 m s^−1^ winds, ECP of 920 hPa, CPD of 84 hPa) again over the ocean. In contrast, Viyaru reached up to a CS level (MSWS of 23 m s^−1^, ECP of 990 hPa, CPD of 10 hPa), and dissipated after landfall as a tropical cyclone. In this study, we showed that all three cyclones were formed in the southeastern Bay, which is rare during 1982–2020. This is related to an increase of a significant SST trend in May (0.02–0.04 °C per year) in that region, possibly due to excessive heat storage during winter and spring in recent years (which needs to be investigated in the future). Favorable atmospheric conditions interacting with the strong northward WBC and its eddies during April–May 2019 and May 2020 might also have helped to intensify both Fani and Amphan^31^, which will require further investigation including coupled numerical model experiments. A quantitative measure of along-Track Available Heat Potential (TRAPH) shows the peaking of cyclone intensity along the path over the WBC and its eddies for Fani and Amphan, which was missing for Viyaru. Our analysis suggests that more frequent occurrence of such high-intensity pre-monsoon cyclones (Fig. 1d) in the Bay of Bengal might be the new normal in the Bay of Bengal in a warming future. This shifting seasonality obviously has deep implications for public safety and hazards, agriculture, and maritime activities in the future.

Finally, it is worth mentioning a couple of possible worldwide implications of our findings. Similar processes can lead to future intensification of north Atlantic storms passing over the Gulf Stream and its warm core rings. Recent observational studies have shown that the warm core ring formation over the Gulf Stream region (75–55° W, 35–45° N) increased from an average of 18 per year during (1980–1999) to 33 per year during (2000–2017)^47,48^. The formation of these warm core rings reaches its peak in August. The implication of such abundance of warm rings at the beginning of the Atlantic hurricane season is unknown currently. Similar implications may apply to the Pacific typhoons crossing the Kuroshio. In addition, Yang et al.^49^ documented poleward shifts of Western Boundary Currents and projected northward shifts and increased poleward heat transport in 2050 and beyond from CMIP6 downscaling experiments^50^. Our findings (and the quantitative approach presented herein), supported by additional refinement with high-resolution data and coupled ocean–atmosphere modeling systems could contribute towards better predictability of the propagation paths of these cyclones and their subsequent amplitude manifestation along-track. In 2021, the VSCS cyclone Yaas formed in the northern Bay of Bengal (16.3° N 89.7° E) in the warm pool region in the northern Bay of Bengal near 18° N (Fig. 2). The formation location was exceptionally warm (by about 2 °C above climatological value), and the path of Yaas went over a large warm eddy area. However, according to IMD report on Yaas^51^, this cyclone never went through rapid intensification during its lifetime, indicating that atmospheric parameters, mainly the wind shear, might have hindered the rapid intensification of this cyclone despite very favorable SSTs. Further studies on this latest cyclone and such competition between atmospheric and oceanic roles in cyclone intensification are needed. To conclude, the evolving extreme severity of hurricanes, typhoons and cyclones can be aided by the warming Western Boundary Currents and their warm eddies in the future climate.

## Methods

### Data (cyclone, SST, SSH, winds)

For the current study, the cyclone tracks, intensity, maximum sustained winds speed, estimated central pressure, and central pressure drops for all cyclones were obtained from the India Meteorological Department (IMD) datasets (https://rsmcnewdelhi.imd.gov.in/). The inter-annual trends of SST were computed from the UK Met Office Hadley Centre’s Sea ice and SST data set^37^ (source: https://climatedataguide.ucar.edu/climate-data/sst-data-hadisst-v11). Trend (°C/year) is calculated at each grid point over the domain and average is calculated. We have performed the student’s two-tailed t-test and identified the regions with a significance level of more than 95% (90%) with p-value less than 0.05 (0.01).

Daily SST data during cyclones were collected from the Global Higher Resolution Sea Surface Temperature (GHRSST) with spatial resolution 1/20° during the season of the cyclones (source: http://apdrc.soest.hawaii.edu/las/v6/constrain?var=12679). Altimeter-derived daily SSHA fields of 1/4° spatial resolution, were extracted from the satellite-derived Archiving Validation and Interpretation of Satellite Oceanographic (AVISO) datasets (source: http://marine.copernicus.eu/). Scatterometer-derived 25-km winds were obtained from SCATSat-1 for Fani (2019) and Amphan (2020) (source: https://www.mosdac.gov.in). Winds for Viyaru (2013) were obtained from ASCAT http://apdrc.soest.hawaii.edu/las/v6/dataset?catitem=12698). The Argo profile (WMO: 2902283) is obtained from https://fleetmonitoring.euro-argo.eu/float/2902283.

### Current intensity (CI) and cyclone stages

The ‘CI’ stands for Current Intensity as given in the best track dataset by IMD (https://rsmcnewdelhi.imd.gov.in/uploads/report/bestrack.pdf). The CI is an instantaneous measure of the strength of the cyclone in wind speed and varies from 1.5 (deep depression stage) to 7 (super cyclone stage) for a cyclone. The stages (wind-speed) of the cyclones are as per IMD: Tropical Depression (TD: 31–50 km/h or 20–31 mph or 17–27 kn), Deep Depression (DD: 51–62 km/h or 32–38 mph or 28–33 kn), Cyclonic Storm (CS: 63–88 km/h or 39–54 mph or 34–47 kn); Severe Cyclonic Storm (SCS: 89–117 km/h, 55–72 mph or 48–63 kn); Very Severe Cyclonic Storm (VSCS: 118–166 km/h or 73–102 mph or 64–89 kn); Extremely Severe Cyclonic Storm (ESCS: 167–221 km/h or 166–221 mph or 90–119 kn); and Super Cyclonic Storm (SuCS: > 221 km/h or > 137 mph or > 119 kn).

### Super cyclone Amphan (May 2020)

Super Cyclonic Storm Amphan was the first tropical cyclone of 2020 in the northern Indian Ocean. It is also the first pre-monsoon super cyclonic storm in recorded history going back to 1991 in the Bay of Bengal. The deadliest cyclone Bhola (1970) that killed over 300,000 people was an Extremely Severe Cyclonic storm (with highest winds of 149 mph)^52,53^ and the Super Cyclone of 1999 that killed around 10,000 people was in the post-monsoon regular cyclone formation season^52,54^. Amphan formed as a depression on 16 May 2020 over exceptionally warm sea surface temperatures of 32–34 °C (observed at 1 m depth from a moored ocean buoy BD13 at 11° N, 86° E; source: https://incois.gov.in/). It rapidly intensified to an extremely severe cyclonic storm on 16 May within 12 h^32^. On 18 May, at 0900 UTC Amphan intensified to the SuCS stage. On the same day, at 1800 UTC its peak intensity with 3-min sustained wind speeds of 240 km/h (150 mph) and a ECP of 920 hPa. On 20 May, between 1000 and 1100 UTC, the cyclone made landfall in West Bengal^55^. See Fig. 2 for its propagation path and intensity evolution along the path. The IMD estimated sustained winds for Amphan to be 155 km/h (85 kn or 100 mph) at landfall. Amphan rapidly weakened once inland and dissipated shortly thereafter. The last super cyclone to hit the city of Kolkata, West Bengal was in October 1737^52^.

### Cyclone Fani (April–May 2019)

The Extremely Severe Cyclone Fani formed from the 26th of April to 3rd May 2019 with one of the longest cyclonic tracks (~ 3030 km) and duration (~ 204 h, i.e., 8 days 12 h) in the southeastern Bay of Bengal and followed a unique track (Fig. 3). Initially, at 0300 UTC of 26th April 2019, Fani formed as depression with MSWS of 13 m s^−1^, ECP of 998 hPa, and a CPD of 4 hPa^46^. Under favorable environmental conditions, and moving northwestwards, it intensified into a deep depression on 0000 UTC of 27th April 2019. It further intensified into a cyclonic storm at around 0600 UTC of 27th April 2019 over the southeast Bay of Bengal. Moving northwestwards, it intensified into a severe cyclonic storm on 29th April 2019 over the southeastern Bay of Bengal with 31 m s^−1^ MSWS, 986 hPa ECP, and 16 hPa CPD at 1200 UTC. It moved northwards and further intensified into a VSCS on 29th April 2019 at 2100 UTC and then into an ESCS on 30th April 2019 at 1200 UTC with 46 m s^−1^ MSWS, 962 ECP and 40 hPa CPD, when it crossed the WBC (Fig. 3). On 3rd May 2019, Fani crossed the Odisha coast close to Puri between 0230 and 0430 UTC with MSWS ~ 51 m s^−1^, ECP ~ 952 hPa and CPD ~ 50 hPa^46^. Continuing to move northeastwards, it weakened into a Very Severe Cyclonic Storm over coastal Odisha and moving further northeastwards weakened into a Severe Cyclonic Storm over north Odisha around 1500 UTC of the same day, ultimately dissipating by 5th May 2019.

### Cyclone Viyaru (May 2013)

The Cyclonic Storm Viyaru was a comparatively weaker Tropical Cyclone, which matured over the central and northwestern Bay of Bengal from 10 to 16th May 2013 (Fig. 1). Initially, a depression formed over the southeast Bay of Bengal at around 0900 UTC of 10th May 2013^34^. Under favorable conditions, it intensified into a Cyclonic Storm at around 0600 UTC of 11th May 2013. Viyaru moved to the northeast on 14th May 2013 and by early morning on 16th May 2013, winds speed significantly increased to ~ 23 m s^−1^, ECP of 990 hPa, and CPD of 10 hPa. Viyaru crossed the Bangladesh coast at 22.8° N, 91.4° E with an MSWS of 23–26 m s^−1^. After the landfall, it continued to move northeastwards and weakened gradually due to interaction with the land surface. It further weakened into a well-marked low-pressure system over Nagaland in the early morning of 16th May 2013 and moved away towards Myanmar^56^.

### Calculation of threshold wind speed for TRAPH

The wind speed distribution within a cyclone is not radially symmetric. Typically, the winds within a cyclone are thought to reach maximum speed some distance away from the center and then weaken out. However, there are patches of stronger winds, or footprints of the high-energy bands within the cyclone before reaching the maximum wind and afterwards and these patches are distributed within the cyclone area depending upon the ocean–atmosphere feedback and energy conversion. It is thus important to identify this ‘energy gathering footprint’ of the cyclone. This is the effective region within the cyclone where the winds gather energy from the ocean. We designed a ‘threshold wind speed’ for a cyclone from the basin-wide wind distribution during the cyclone passage and calculated the area covered by the winds bound by the threshold on either side of the maximum wind speed to determine the ‘energy gathering footprint’ of the cyclone. The threshold value also helps capture the relative strength of the cyclone and its growth compared to its associated surrounding large scale wind field over the Bay of Bengal, which differs for different cyclones.

**Figure 6:**
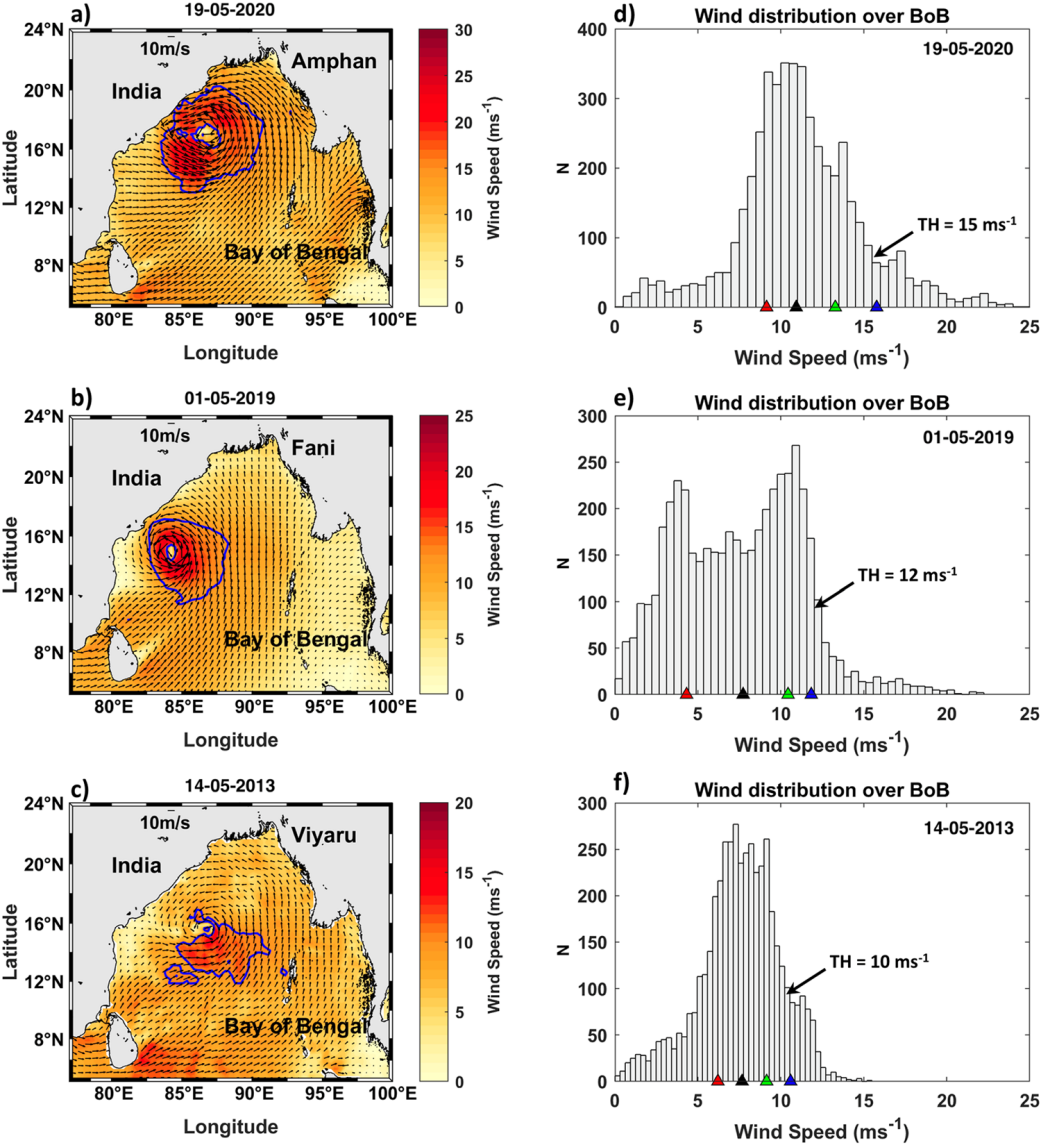
Wind speed (shaded color, ms^−1^) for (**a**) Amphan, (**b**) Fani and (**c**) Viyaru with wind directions. Histogram for wind speeds over the Bay of Bengal (4–24° N, 76–100 $^\circ$ E) for three cyclones on selected days. Red, black, green, and blue dots indicate the 25th, 50th, 75th and 90th percentile. ‘N’ denotes the number of grid points. Blue contours on the left panels are for the threshold wind speeds (TH on the right) in (**a**) 15 m s^−1^, (**b**) 12 m s^−1^, and (**c**) 10 m s^−1^. The relative strength of the three cyclones are visible in the wind field on the left as well as in their domain-wide distribution on the right. The maps (coastlines) are created with Matlab M_Map v1.4 toolbox (https://www.eoas.ubc.ca/~rich/map.html).

The spatial distributions of winds for 19th May 2020 (Amphan), 1st May 2019 (Fani), and 14th May 2013 (Fani) show the asymmetries in the winds around the center of the cyclone (Fig. 6a–c). Amphan showed the maximum wind speed on the southwest side of the center (Fig. 6a), whereas it is on the southeast for Fani (Fig. 6b). The winds are much weaker for Viyaru, however, the maximum winds are seen on the southeast of the center. Therefore, the histogram of the winds over the whole of Bay of Bengal is analyzed for identifying the threshold value of the winds with higher magnitude. A single-peak modal distribution was found for Amphan with magnitude more 10 m s^−1^ in the whole Bay of Bengal, indicating the impact on the larger area. For Fani, there are two peaks. The peak with lower wind magnitude around 5 m s^−1^ indicates the ambient seasonal winds. The other peak with higher magnitude of 10 m s^−1^ showed the influence of cyclone. In case of Viyaru, there is also a single peak with wind magnitude of 7 m s^−1^ showed the impact area is much less and mostly influenced by the seasonal winds.

The distribution of winds over the Bay of Bengal for the three cyclones on selected days are shown in Fig. 6d–f. Our approach was to choose the threshold wind speed by using a percentile categorization of the observed wind distribution. A sensitivity test was carried out with different percentile limits for determining the threshold. It was clear that the threshold defined by the 90th percentile wind speed captured most of the energetic footprint around the center similar to Figs. 3, 4 and 5 and as shown in Fig. 6a–c. The footprint signature with wind speeds given by the 80th or 70th percentile captured more wind patches outside and away from the cyclone area, which was rendered undesirable. Thus, we chose the 90th percentile values of the wind speeds (Amphan 15.7 m s^−1^, Fani 11.8 m s^−1^ and Viyaru 10.6 m s^−1^) as representative of the high wind impact areas for all three cyclones. Therefore, wind thresholds are taken as 15, 12 and 10 m s^−1^, respectively. The grids with wind speeds over the threshold values are identified, and the SST values at those grids are used for the calculation of the TRAPH.

Finally, note that this approach has at least three important attributes: (i) it appreciates the importance of stronger patchiness within the cyclone; (ii) it eliminates the weaker winds near the center low; and (iii) it captures the areas of stronger winds beyond the maximum wind radius as well.

## Data Availability

The datasets generated during and/or analyzed during the current study are also available from the corresponding author on reasonable request.
